# Prevalence of microalbuminuria and diagnostic value of dipstick proteinuria in outpatients from HIV clinics in Bukavu, the Democratic Republic of Congo

**DOI:** 10.1186/1471-2369-15-146

**Published:** 2014-09-05

**Authors:** Mannix Imani Masimango, Ernest Kiswaya Sumaili, Michel Jadoul, Pierre Wallemacq, Dieudonné Kanigula Mubagwa, Rissassy Jean-Robert Makulo, François Bompeka Lepira, Nazaire Mangani Nseka

**Affiliations:** Department of Medicine, Université Catholique de Bukavu, Bukavu, DR Congo; Nephrology Unit, Department of Medicine, Université de Kinshasa, Kinshasa, DR Congo; Department of Nephrology, Cliniques Universitaires Saint-Luc, Université catholique de Louvain, Brussels, Belgium; Clinical Chemistry Department, Cliniques Universitaires St Luc, Louvain Center for Toxicology And Applied Pharmacology, Université Catholique de Louvain, Brussels, Belgium; Department of Cardiovascular Sciences, KU Leuven, Leuven, Belgium; Postal address: 103, Cyangugu, Rwanda

**Keywords:** Dipstick proteinuria, Albumin/creatinine ratio, Diagnostic value, HIV-patients

## Abstract

**Background:**

Microalbuminuria is a marker of early kidney disease and high cardiovascular risk in various populations, including HIV positive patients. However, the diagnostic value of qualitative (dipstick) proteinuria and the burden of microalbuminuria in HIV positive patients living in sub-Saharan Africa are relatively unclear.

**Methods:**

In a cross-sectional study, 235 HIV- positive outpatients were screened for proteinuria in 3 HIV-clinics in Bukavu. A spot urine sample from each subject was tested both by a dipstick and albumin-creatinine-ratio (ACR) assay. The performance of dipstick proteinuria exceeding 1+ was compared with that of microalbuminuria (≥30 mg/g creatinine).

**Results:**

The prevalence of microalbuminuria and dipstick proteinuria ≥ (1+), ≥ (2+) and ≥ (3+) was 11%, 41%, 3.5% and 0.7%, respectively.

Compared to microalbuminuria, the dipstick (proteinuria of 1+ or greater) had an overall sensitivity of 60% and a specificity of 61%. The positive predictive value was 15.4% and the negative predictive value 92.8%.

**Conclusion:**

Proteinuria is highly prevalent in HIV positive patients. The limited sensitivity and specificity of the dipstick to detect significant microalbuminuria make it unattractive as a screening tool in HIV positive patients.

## Background

The prevalence of chronic kidney disease (CKD) is high in the African HIV infected population [1, 2] where late referral to hospital and scarce possibilities of renal replacement therapy are well known. The early detection of CKD would provide the opportunity to implement strategies known to slow CKD progression [3, 4]. Thus, the identification of overt proteinuria or even microalbuminuria, might be an important tool for the early detection of CKD in HIV-infected individuals [5, 6]. The Infectious Diseases Society of America (IDSA) guidelines on the management of chronic kidney disease in HIV-positive patients recommend the use of a urine dipstick, with a threshold for detection of renal disease at 1+ proteinuria. When dipstick is ≥ 1, a quantification of proteinuria is recommended. Currently, this relies on the albumin/creatinine ratio (ACR) measured on a spot of urine [7, 8]. However, in many emerging countries, the dipstick remains the only method used for the evaluation of proteinuria. Nevertheless, it is recognized that dipsticks may have poor sensitivity in detecting low-grade proteinuria [9].

The purpose of the current study was to evaluate the prevalence of proteinuria in HIV-positive patients and to assess the diagnostic value of urinary dipstick as screening test for proteinuria compared with the albumin/creatinine ratio (ACR).

## Methods

Written informed consent for participation in the study was obtained from participants.

A cross-sectional survey was conducted in March and April 2012 in 3 outpatient HIV-clinics in Bukavu, the main town in the eastern rural part of the Democratic Republic of Congo (DRC).

Two hundred and sixty consecutive HIV-1 positive adults (≥18 years) who routinely attended one of the clinics during the survey period were screened.

Single spot urine specimens were collected in the morning. Urine samples containing blood (1+ or greater), white blood cells (1+ or greater) and/ or nitrites were excluded. This left 235 subjects in whom, both a urine dipstick test and a urine albumin to creatinine ratio measurement were performed (ACR). The results of urine dipstick (Multistix 8 SG®, Siemens Healthcare Diagnostics, France) were read as either negative (<30 mg/dl) or positive at 1+, 2+, 3+ or greater, corresponding approximately to protein concentrations of 30, 100 mg/dl , 300 and ≥ 2000 mg/dl. ACR was performed using the DCA Vantage^TM^ analyzer (Siemens Healthcare Diagnostics Pyt Ltd., 885 Mountain Highway, Australia), utilizing an immunoassay method for Albumin. Microalbuminuria was defined by a urinary albumin-to-creatinine ratio (ACR) 30–299 mg/g and macroalbuminuria by an ACR > 300 mg/g. The diagnostic value (sensitivity, specificity, positive predictive value, negative predictive value) of dipstick ≥ 1+ and ≥ 2 + to detect micro or macro albuminuria was thus assessed.

### Statistical tools

The processing and data analysis were performed using SPPS software Windows version 18.0 and MedCalc 9.2.1.0.

Quantitative albuminuria expressed as ACR was subdivided into three sub-groups: <30 mg/g (normoalbuminuria), 30 to 299 mg/g (microalbuminuria) and ≥ 300 mg/g (macroalbuminuria), and compared with the respective results of the dipstick on the same sample.

The diagnostic value (sensitivity, specificity, positive predictive value, negative predictive value) of the dipstick 1+ or greater (>30 mg/dl) was thus studied. The cutoff of 1+ was chosen on the basis of IDSA’s recommendations.

### Ethical issues

Permission obtained from the Ethics Committee of the school of Medicine of the Catholic University of Bukavu (Commission Institutionnelle d’Ethique, 09 juin 2012).

## Results

A total of 260 patients were contacted. Twenty-five had to be excluded because of hematuria (n = 12), detection of nitrites in urine (n = 8) or leucocyturia (n = 5). The 25 excluded patients had demographic characteristics similar to those of the 235 included patients.

A total of 235 patients were thus included, of whom 179 (76.2%) were on antiretroviral therapy. Baseline data are provided in Tables 1, 2 and 3. All patients were African. Median age was 40 years (IQR 18–74) and 73.2% were women.

**Table 1:** **General characteristics of studied population**

Variables	Whole group	HAART subjects	ART naive subjects	p
	n = 235	n = 179	n = 56	
Female, %	172 (73,2)	127 (54)	45 (19,1)	0,0001
Age (years, median)	40,0 ± 10,7	42,3 ± 10,6	35 ± 9,7	< 0,0001
Family history of:				
- DM	48 (20,4)	41 (17,4)	7 (3)	0,06
- Hypertension	72 (30,6)	54 (23,0)	18 (7,7)	0,28
- Obesity	50 (21,3)	39 (16,6)	11 (4,7)	0,61
- CKD	22 (9,4)	15 (6,4)	7 (3,0)	0,49
Comorbidities				
- DM	4 (1,7)	4 (1,7)	0 (0)	0,597
- Hypertension	110(46,8)	87 (37,0)	23 (9,8)	0,02
Level of education < 6 years	113 (48,9)	79 (34,2)	34 (14,7)	0,05
Marital status:				
- Married	104 (55,7)	78 (43)	26 (12,8)	0,01
- widow	131 (55,7)	101 (43)	30 (12,8)	0,005
History of smoking	28 (11,9)	21 (8,9)	7 (3)	0,73
History of alcohol consumption	107 (45,5)	82 (34,9)	25 (10,6)	0,036
History of medicinal plants use	29 (12,3)	19 (8,1)	10 (4,3)	0,69
History of NSAIDS use	49 (20,9)	39 (16,6)	10 (4,3)	0,62

Data are expressed as absolute or relative frequencies or mean plus the standard deviation (SD) as appropriate.

**Table 2:** **Clinical characteristics of subjects**

Variables	Whole group	HAART subjects	ART naive subjects	p
	n = 235	n = 179	n = 56	
Known HIV infection duration (years)	4,7 ± 3,2	5,3 ± 3,04	2,8 ± 2,9	<0,0001
Duration of HAART (months)	NA	46,2 ± 30,08	NA	
WHO stage for HIV clinical disease:				
- Stage 1	25 (10,6)	12 (5,1)	13 (5,5)	0,39
- Stage 2	53 (22,6)	21 (8,9)	32 (13,6)	0,93
- Stage 3	140 (59,6)	129 (54,9)	11 (4,7)	0,0039
- Stage 4	17 (7,2)	17 (7,2)	0	<0,0001
Body mass index (kg/m^2^)	22,3 ± 3,8	22,5 ± 3,9	21,7 ± 3,4	0,198
SBP (mmHg)	118,82 ± 20,89	120,58 ± 21,7	113,2 ± 16,7	0,023
DBP (mmHg)	77,7 ± 14,02	79,8 ± 14,7	71,8 ± 11,6	0,0002
HAART regimen:				
AZT + 3TC + NVP	NA	164 (94,8)	NA	
d4T + 3TC + NVP	NA	3 (1,6)	NA	
ABC + DDI + Lp/r	NA	9 (5)	NA	
TDF + 3TC + EFV	NA	3 (1,6)	NA	

HTA = hypertension; BMC = body mass index; SBP = Systolic blood pressure; DBP = diastolic blood pressure; CKD = chronic kidney disease; HAART: Highly active antiretroviral therapy. NA: not applicable; d4T: stavudine, 3TC: lamivudine, NVP: nevirapine, ABC: abacavir, DDI: didanosine, Lp/r : lopinavir/ritonavir, EFV: efavirenz, TDF: tenofovir.

**Table 3:** **Biological characteristics of subjects**

Variable	Whole group	HAART group	ART naïve group	p
Fasting glycaemia	103,5 ± 14,4	104,2 ± 15,1	100,3 ± 10,6	0,27
Hemoglobin	13,3 ± 2,0	13,5 ± 1,9	12,7 ± 2,2	0,01
HBs Ag positive	5 (1,9)	5 (2,1)	0 (0)	0,44
Anti HCV positive	3 (1,2)	1 (0,4)	2(0,9)	<0,0001
CD4 (cells/mm^3^ increment):				
- < 200	22 (10,7)	14 (6,8)	8 (3,9)	0,50
- 200-499	96 (46,8)	80 (39)	16 (7,8)	0,034
- > 500	87 (42,4)	66 (32,2)	21 (10,2)	0,089

Variables are absolute numbers and relative frequencies or means plus the standard deviation (SD) as appropriate. CD4: cluster of differentiation.

Median CD4 cell count was 480 cells /mm3 (IQR 10–1202). The overall prevalence of proteinuria according to ACR was 10.6% (95% CI 6.67- 14.53), 8.9% for microalbuminuria (ACR 30–300 mg/g) and 1.7% for macroalbuminuria (ACR >300 mg/g). In contrast, the prevalence of dipstick urine protein (1+), (2+) and (3+) were 37%, 3.4% and 0.9%, respectively.

Regarding the intensity of proteinuria, no difference was observed between the groups receiving antiretroviral versus ART naive patients.

The 235 paired dipstick and quantitative results are displayed in Table 4.

**Table 4:** **Comparison between the dipstick and the ACR in 235 patients**

		A/C ratio (mg/g)		
Urine dipstick	<30	30-299	≥ 300	Total
	n = 210	n = 21	n = 4	n = 235
0	128	10	0	138
1+	76	10	1	87
≥ 2+	6	1	3	10

(*) The numbers with gray shading correspond to false-positive dipstick results. The numbers highlighted in orange are false-negative ones.

Seventy-six (36.2%) of 210 patients with urine ACR < 30 mg/g had a positive urine dipstick results (false-positive result ≥ 1+). Furthermore, 10 (47.6%) of 21 patients with abnormal low-level albuminuria (UACR 30 to 299 mg/g) had negative urine dipstick results (false-negative result: 1+).

In comparison with ACR > 30 mg/g, urine dipstick had an overall sensitivity and specificity of 60% and 61%, respectively. At a prevalence of proteinuria of 10.6%, the positive predictive value of positive urine dipstick for ACR >30 mg/g was 15.4% and the negative predictive value 92.8%.

## Discussion

The purposes of this study were to determine the prevalence of microalbuminuria in an HIV-infected clinic population in DRC and the performance of the dipstick urine test compared to the ACR. Approximately three quarter of the subjects were women, consistent with other data showing a feminization of HIV infection, particularly in Africa [10, 11].

In the present study, the prevalence of proteinuria with dipstick test (≥1+) was 41.3%. Cohort studies suggest that approximately 30% of HIV-positive individuals have proteinuria (≥1+). Similar results are reported by Fabian *et al*. in South Africa (44%) [12] and Jao *et al.* in Cameroon (39%) [13]. However, Struik *et al.* [14] and Longo *et al.* [15] reported a lower prevalence, 23 and 21% respectively. Furthermore, the prevalence of quantitative proteinuria defined as ACR ≥ 30 mg/g was 10.6%. This prevalence is similar to that of earlier reports varying from 6% to 30% in the HIV-infected, which is three to five times higher than in the general population [16–19]. Differences in methodological approach and in clinical profile of patients could probably explain the variability of results between studies.

Although, this study did not address the specific causes of proteinuria in individual subjects, it is likely that proteinuria is related to HIV itself. Experimental studies demonstrate a direct impact of viral components (gp120, TAT) on the endothelium, as they lead to the expression of adhesion molecules (intercellular adhesion molecule [ICAM], E-selectin), a prothrombotic state (increase of von Willebrand factor, plasminogen activator inhibitor-[PAI-]1, tissue plasminogen activator [t-PA], tissue factor). In addition, soluble adhesion molecules (selectin, ICAM) were found to be increased in HIV-infected persons [20]. HIV-associated nephropathy (HIVAN) is the commonest biopsy finding among patients with HIV infection who present with proteinuria. Han *et al.* reported that 25% of naïve HIV-positive black patients (without hypertension, diabetes and with serum creatinine < 250 μmol/L), have microalbuminuria, and that HIVAN was found in 86% of the subset of such patients with persistent micro albuminuria [19].

The results of our study suggest that the urine dipstick may not be an adequate tool for screening for proteinuria in HIV-positive patients. As a screening test, the sensitivity (60%) and specificity (61%) of a single dipstick is poor. In fact, at lower levels of proteinuria (30 to 299 mg/g), a threshold of 1+ dipstick had a false-negative rate of 47.6%. Therefore, nearly 1 in 2 patients with proteinuria in this range may not be recognized and may have delayed workup and treatment. In addition, the proportion of false positives was 36%. Consequently, one to 3 patients with positive dipstick (1+) could be falsely considered to be at high cardiovascular and kidney risk.

Several studies reported the poor sensitivity of dipstick testing to identify early renal disease in HIV-population [9], hypertensive [21] and diabetic patients [22]. A recent study has demonstrated that the sensitivity of dipstick test, may be affected by urinary concentration [9]. In addition, dipstick tests may miss about one out of five people with kidney disease, and positive dipstick test results for proteinuria may have to be confirmed by other laboratory tests [23]. In fact, in a study of 166 HIV-infected persons who underwent both a urine dipstick and a spot urine protein-to-creatinine ratio within a 48-hour period, 21% of those with low but overt proteinuria (300 to 900 mg/g creatinine) were not detected by urine dipstick. So, in individuals at renal risk, such as HIV + patients, the search for microalbuminuria is recommended when the dipstick is negative. Furthermore, the recent Kidney Disease Initiative and Global Outcome (KDIGO) CKD Guidelines recommend, in individuals with proteinuria by dipstick, to perform confirmatory protein quantification within 3 months [24]. Defining proteinuria as a positive urine dipstick test on two occasions [25] could have reduced the false positive rate in our study, but probably at the expense of an even higher false negative rate.

## Conclusion

### Strengths of the study

Because of the high prevalence of proteinuria, early screening efforts should be encouraged in HIV-patients both before and under antiretroviral treatment. Based on the accuracy of the semi-quantitative urine dipstick, the KDIGO CKD Guidelines suggest the use of either a spot protein-to-creatinine or spot albumin-to-creatinine ratio for kidney disease screening [24]. The results of the current study question the use of the urine dipstick as screening tool for low-level proteinuria in HIV patients. Although dipstick testing would decrease costs, it would fail to diagnose most patients with microalbuminuria, an early marker of glomerular injury. Therefore, HIV + patients with negative dipstick should be screened for microalbuminuria. The Infectious Diseases Society of America (IDSA) guidelines that recommend using the dipstick as a screening tool may need to be revisited and updated.

### Limitations of the study

It is a cross-sectional study of consecutively recruited patients with a single measurement (serum creatinine and proteinuria) and a relatively small sample size, thus limiting the generalization of the results to the entire population of patients living with AIDS. In addition, patients naive to ART were also underrepresented limiting the generalization of results obtained.
